# Metal-Organic Framework-Derived NiSe Embedded into a Porous Multi-Heteroatom Self-Doped Carbon Matrix as a Promising Anode for Sodium-Ion Battery

**DOI:** 10.3390/nano12193345

**Published:** 2022-09-26

**Authors:** Xiaoyan Shi, Lujun Fang, Handong Peng, Xizhan Deng, Zhipeng Sun

**Affiliations:** School of Materials and Energy, Guangdong University of Technology, Guangzhou 510006, China

**Keywords:** sodium-ion batteries, nickel selenide/carbon composites, metal–organic framework, heteroatom doping

## Abstract

A self-doping strategy is applied to prepare a multi-heteroatom-doped carbonaceous nickel selenide NiSe@C composite by introducing N and P-containing ligand hexa(4-carboxyl-phenoxy)-cyclotriphosphazene (HCTP-COOH) into a Ni-based MOF precursor. The MOF-derived NiSe@C composite is characterized as NiSe particles nested in a multi-heteroatom-doped carbon matrix. The multi-heteroatom-doped NiSe@C composite with a unique structure shows an excellent sodium-ion storage property. The Na-ion battery from the NiSe@C electrode exhibits a capacity of 447.8 mA h g^−1^ at 0.1 A g^−1^, a good rate capability (240.3 mA h g^−1^ at 5.0 A g^−1^), and excellent cycling life (227.8 mAh g^−1^ at 5.0 A g^−1^ for 1200). The prospects of the synthesis methodology and application of NiSe@C in sodium-ion batteries (SIBs) devices are presented.

## 1. Introduction

The study of sodium-ion storage has been under the spotlight due to its lower cost and more abundant resources of sodium when compared to lithium [1,2,3]. Additionally, dramatic volume changes in the electrodes of sodium-ion batteries (SIBs) caused by the insertion/desertion of large Na^+^ ions have slowed their practical application severely [4,5,6,7,8,9]. Therefore, the design and synthesis of stable electrodes with tolerable volume changes have been the key tasks for improving the electrochemical performance of SIBs. Among those, metal selenides have attracted enormous attention because of their high-rate performance and comparatively good cycling performance resulting from their high electronic conductivity and structural stability [10,11,12]. Noticeable volume variations and dramatic aggregation are still inevitable during long-term cycling, which results in suboptimal cycle performance for the metal selenides [13,14,15].

Fabricating corresponding carbonaceous composites is known as an efficient method to achieve metal selenide-based electrodes with better stability. The carbon matrixes can generally restrict the volume changes of the metal selenides therein to enhance the structural stability and improve the conductivity of the whole composites at the same time [16,17,18]. A straightforward strategy to prepare these composites is from the metal–organic frameworks (MOFs) [19,20]. The calcination or pyrolysis treatment of metal- and organic-ligand-composed MOFs derived materials has shown impressive electrochemical properties in different energy storage devices [21,22,23]. This is due to the metal-phased electroactive material being enwrapped with the in situ generated carbon matrixes by calcination. As mentioned above, this structure could remarkably alleviate the volume expansion during repeated sodiation/desodiation cycles and simultaneously improve electronic conductivity [20].

It is acknowledged that heteroatom doping (i.e., N, P, O, and B) into the electrode materials can boost their electrochemical performance [24]. For MOF-derived material, the introduction of N or P heteroatoms can be readily realized using N- or P-containing ligands during the synthesis of its MOF precursor [25,26]. However, in general, to obtain multi-heteroatom doping composites, several synthetic steps are needed. Meanwhile, MOFs-derived metal selenides/multi-heteroatom self-doped carbon are seldom explored for SIBs. In light of the information above, here, a porous composite composed of NiSe embedded in multi-heteroatom (N, P, and O) self-doped carbon matrix (NiSe@MC) was successfully synthesized through a facile strategy from a Ni-based MOFs precursor containing ligand hexa(4-carboxyl-phenoxy)-cyclotriphosphazene (HCTP-COOH). The usage of the HCTP-COOH ligand realized the self-doping of multi-heteroatom (N, P, and O) in one carbonization step. Furthermore, the electrochemical performance of as-prepared NiSe@MC in SIBs was investigated and excellent properties were observed. This work proved the applicability of MOFs containing HCTP-COOH ligands in deriving multi-heteroatom self-doped composites and their applications in sodium storage devices.

## 2. Materials and Methods

### 2.1. Preparation of NiSe@C Composites

The Ni-MOF was synthesized by a typical hydrothermal method. First, the HCTP-COOH (399 mg) ligand and C_4_H_6_O_4_Ni·4H_2_O (320 mg) were dissolved in 35 mL of DMF, and the mixture was stirred at room temperature for five minutes; then, the light-green precipitate was isolated from the solution by centrifugation and further washed with DMF and THF several times, and it was further dried at 70 °C overnight.

To obtain the Ni@C, 1 g Ni-MOF was annealed at 600 °C for 2h in an Ar atmosphere with a rate of 1 °C min^−1^.

To prepare the NiSe@C, 0.316 g Se powder was dissolved in 10 mL 80% N_2_H_4_·H_2_O and stirred for 10 min, then 0.18 g Ni@C and 50 mL H_2_O were added into the solution and stirred for another 30 min. Then, the mixture was transferred into a 100 mL Teflon-lined autoclave and maintained at 200 °C for 12 h. The synthesized products were washed with distilled water and ethanol three times. Then, the products were dried at 80 °C for 10 h in a vacuum oven and then annealed at 600 °C for 2 h under an Ar atmosphere.

### 2.2. Materials Characterization and Electrochemical Measurements

Detailed information on material preparation and electrochemical property investigation for the as-mentioned materials is included in the electronic Supporting Information File.

## 3. Results

**Figure 1:**
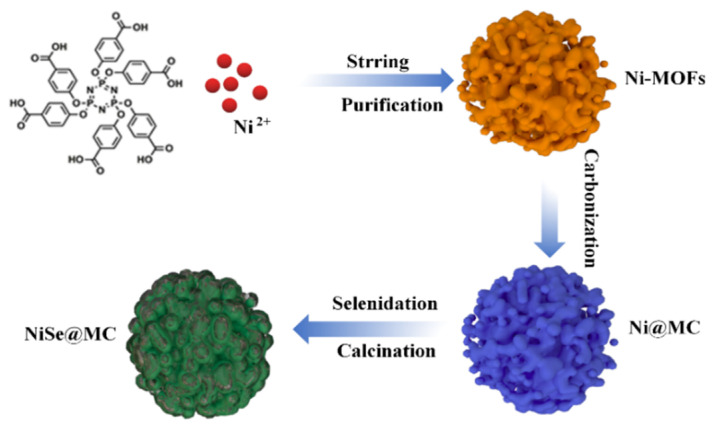
Illustration of the synthesis procedure of NiSe@MC composites.

Figure 1 presents the synthesis process of NiSe@MC. The reaction of Ni(CH_3_COO)_2_∙4H_2_O and HCTP-COOH (molar ratio of 3:1) provided the Ni-MOFs precursor, annealing the Ni-MOFs at 600 °C, followed by a selenization procedure, which resulted in the final product NiSe@MC composites; these were further proven to be multi-heteroatom-doped carbon-matrix-wrapped nickel-selenide composites by various techniques.

**Figure 2:**
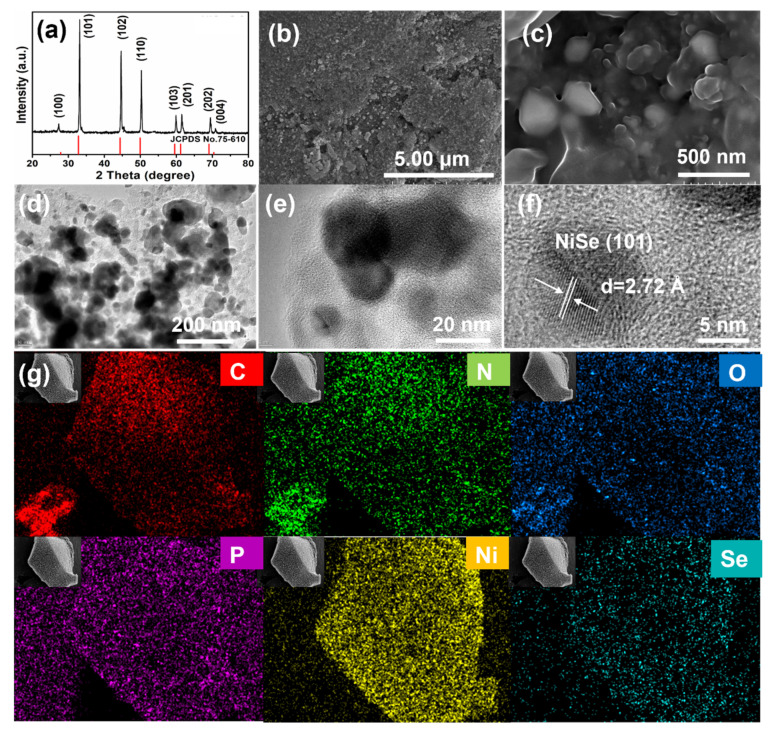
Characterization of NiSe@MC composites: XRD pattern (**a**), SEM images (**b**,**c**), TEM images (**d**–**f**), and EDS analysis (**g**): C (red), N (green), O (blue), P (purple), Ni (yellow), Se (cyan).

The morphology of the precursor and samples after selenization was characterized by a scanning electron microscope (SEM), as shown in Figure 2b,c and Figure S1. The Ni-MOFs precursor shows the nanoparticle shapes of the MOFs pristine; however, many irregular nanoparticles aggregated and formed the bulk material with a smooth surface (Figure S1a,b). Ni@MC presents as a porous bulk material with a relatively rough surface, which is composed of many irregular nanoparticles with a diameter of about 120 nm (Figure S1c,d). NiSe@MC also shows a similar morphology, while the surface of the composites appears to be rougher and more porous (Figure S1e,f). At higher magnification (Figure 2c), the observed nanoparticles display diameters ranging from 100 to 250 nm and are embedded in the bulk carbon materials. This was further proven by the transmission electron microscope (TEM) technique. As observed in Figure 2d,e, abundant dark and irregular particle-like nanostructures with diameters of 20–200 nm can be observed in the grey carbon matrix. Meanwhile, these irregular particles are coated with carbon, which indicates that carbon matrices surrounding the NiSe core can obviously protect the NiSe from harsh environments and effectively enhance conductivity. A crystal lattice with the interlayer spacing of 2.72 Å presenting in high-resolution TEM can be assigned to the crystal plane of (101) of NiSe (Figure 2f). To study the crystallographic information of NiSe@MC composites, X-ray diffraction (XRD) analysis was performed (Figure 2a). The main diffraction peaks at 32.8°, 44.4°, 49.8°, 59.6°, 61.4°, and 68.9° can be ascribed to the (101), (102), (110), (103), (112) and (202) planes of NiSe (JCPDS card no.75-610), respectively, proving that the NiSe phase was successfully prepared by this in situ selenization of Ni-MOFs. Furthermore, the EDS spectrum (Figure 2g) further displays that the elements of C, N, O, P, Ni, and Se are uniformly distributed throughout the whole sample. The atomic ratio of Ni: Se is close to 1:1, suggesting a right molar composition ratio of NiSe. Overall, the results prove that the product NiSe@MC is a multi-heteroatom co-doped carbonaceous nickel selenide. As shown in Figure S2, the thermogravimetric analysis (TGA) result shows 51.3 wt% weight loss from 200 to 750 °C in the air caused by the formation of NiO and the removal of carbon, according to the oxidation product of NiSe@MC in air. As a result, the percentage for NiSe in NiSe@MC is estimated to be 89.7%. Moreover, the synthesized NiSe@MC composites present a typical IV sorption behavior observed in the larger pressure range of ca. 0.45–1.0 P/P_0_ (Figure S3a), suggesting multi-modal and hierarchical porosity, i.e., with micro-pores together with mesopores, which are further verified by the pore size distribution displayed in Figure S3b. The porous properties of NiSe@MC composites are summarized as follows: BET surface area is 318.8 m^2^ g^−1^, pore volume is 0.33 cm^3^ g^−1^, and the average pore size distribution is around 0.8 nm, 2 nm, and 20 nm, respectively. It is indicated that large surface areas and abundant micro-pores, together with mesopores, may be beneficial for fast charge/mass transportation, as well as providing rich surface-active sites for reaction.

**Figure 3:**
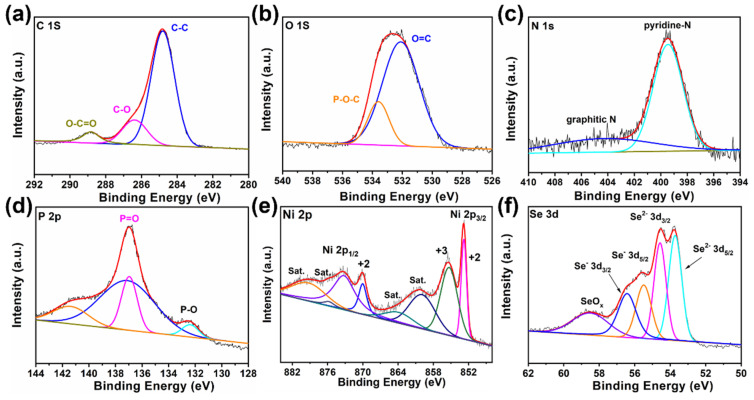
(**a**–**f**) XPS spectra of NiSe@MC: (**a**) C 1s, (**b**) O 1s, (**c**) P 2p, (**d**) Ni 2p, (**e**) N 1s, and (**f**) Se 3d.

The elemental compositions of Ni-MOFs, Ni@MC, and NiSe@MC were primarily studied via EDS analysis (Figure S4). It can be seen that both Ni@MC and NiSe@MC composites inherited the multi-heteroatom elements from the Ni-MOF precursor. The composition and oxidation states of the elements in the NiSe@MC composite were further characterized by X-ray photoelectron spectroscopy (XPS) techniques. The presence of elements C, O, P, N, Ni, and Se in the composite was testified by presenting corresponding characteristic peaks in the XPS survey spectrum (Figure S5), which is consistent with EDS analysis. The doping form of O in the composite is proved to be C-O and O-C=O by both the fine spectra of C 1s and O 1s (Figure 3a,b) [24,25,26]. Moreover, the detailed N 1s spectrum shows two binding energies at 399.4 and 404.1 eV (Figure 3c), which are probably due to the existence of pyridine-N and graphitic N, respectively [27]. In Figure 3d, the existence of P atoms in form of P-O and P=O bonds could be testified through the presence of two peaks at 132.4 and 136.9 eV, respectively, in the detailed spectrum of P 2p. Particularly, two sets of peaks at 852.8/870.0 and 855.4/873.5 eV presented in the fine spectrum of Ni (Figure 3e) could be attributed to Ni^2+^ and Ni^3+^, respectively [28,29]. In Figure 3f, the fine spectrum of Se 3d shows the presence of Se^2−^, Se^−^, and oxidized Se in NiSe@MC composite. The doublet at 55.5/56.4 eV can be assigned to Se 3d_5/2_ and Se 3d_3/2_ of Se^−^, respectively. In addition, the presence of Se^2−^ can be responsible for the other doublet at 53.7/54.6 eV. Moreover, the oxidized Se can be responsible for the peak at 58.72 eV [30,31,32].

**Figure 4:**
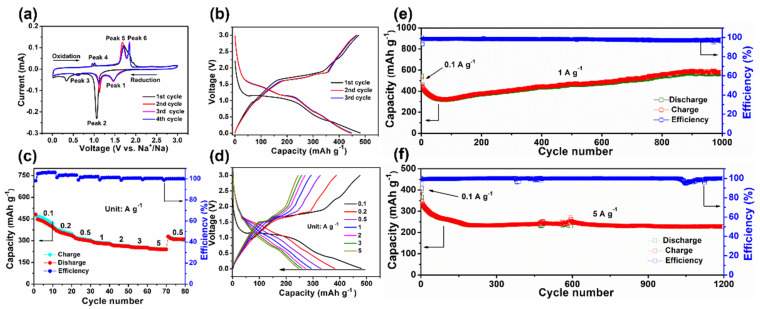
Electrochemical performance of NiSe@MC: (**a**) the first three CV curves, (**b**) the first three GCD cycles, (**c**) rate capability, (**d**) the GCD cycles at various current densities, (**e**) cyclic stability at 1 A g^−^^1^, (**f**) cyclic stability at 5 A g^−1^.

The sodium storage capabilities of the as-prepared carbonaceous nickel selenide NiSe@MC were evaluated by fabricating it into a coin-type sodium-ion half-cell. Figure 4a reveals the first three CV curves of NiSe@MC measured at 0.1 mV s^−1^ (0.01~3.0 V vs. Na/Na^+^). The area difference between the first cycle and the following cycles is the irreversible capacity loss, which can be mainly attributed to the irreversible decomposition of electrolytes and the formation of a solid–electrolyte interface (SEI) layer [27]. Moreover, there are two main reduction peaks after the first cycle, indicating that sodium intercalation is decomposed into two processes. The same is true for the deintercalation processes [4,15]. Figure 4b presents the voltage profiles of NiSe@MC composite. The mismatch between initial and followed charge/discharge profiles is mostly due to the activation of NiSe and side reactions that make the initial sodiation/desodiation process unstable [19]. The following charge/discharge profiles show excellent stability with almost overlap curves and present stable discharge plateaus at 1.46, 1.12, 0.63 V and charge plateaus at 1.02, 1.70, and 1.84 V, indicating good stability of these electrodes. The rate capabilities of the NiSe@MC electrode are shown in Figure 4c. As the current density increases from 0.1 to 0.2, 0.5, 1.0, 2.0, and 5.0 A g^−1^, the discharge capacity of NiSe@MC changes accordingly from 447.8 to 382.0, 329.4, 293.8, 272.9, 257.9, 246.6 and 240.3 mAh g^−1^, respectively. When it is reset to 0.5 A g^−1^ after 70 cycles, the discharge capacity can recover to 310.6 mAh g^−1^, indicating the outstanding high-rate performance of NiSe@MC. The excellent rate capacity could also be evidence that the voltage profiles at the different current densities present identical plateaus. As tested for the cycling performance for 1000 cycles at 1 A g^−1^ (Figure 4e), NiSe@MC exhibits long cyclic stability. The capacity experiences a beginning decay and subsequent increase, which might be caused by the original structural collapse and subsequent pulverization of NiSe particles. The pulverization of NiSe particles could lead to the transformation from the faradaic charge transfer process to interface energy storage, and therefore a gradual capacity increase [33,34]. Even the cycling performance was tested at 5.0 A g^−1^ (Figure 4f), a huge decrease in capacity from 367.3 to 234.2 for the first 200 cycles was observed, probably due to the partial structure damage. After that, the capacity was almost kept unchanged (227.8 mAh g^−1^) until 1200 testing cycles, indicating outstanding cyclic stability at high current density as well. Moreover, NiSe@MC keeps a coulombic efficiency of above 98% during the long-term cycling tests, indicating excellent energy conversion efficiency. The performance of NiSe@MC as an anode for SIBs is compared with previously reported analogues. The superior electrochemical properties proved the applicability of the NiSe@MC composite in sodium-ion storage applications (Table S1).

## 4. Conclusions

In summary, Ni-MOFs-derived multi-heteroatom self-doped NiSe@MC composites were fabricated via a facile strategy. The as-prepared NiSe@MC composites deliver a high specific capacity, long cycle stability, and high-rate performance while used as anodes in SIBs. The porous carbonaceous nickel selenide structure provides protection against volume changes, shortens the charge transfer paths, and improves the utilization efficiency of the active materials. In addition, the self-doping of multi-heteroatom in the carbon matrix could boost the sodium-ion storage behavior of the composites. These results demonstrate that the combination between the use of HCTP-COOH ligand containing MOFs to derive multi-heteroatom self-doped carbons and a further selenization process can afford a straightforward synthetic methodology for the construction of metal selenide nanocomposites, which could be a promising anode for SIBs.

## Data Availability

The data presented in this study are available on request from the corresponding authors.

## Supplementary Materials

The following supporting information can be downloaded at: https://www.mdpi.com/article/10.3390/nano12193345/s1, Figure S1: SEM images of Ni-MOFs, Ni@MC, and NiSe@MC; Figure S2: TGA curve of NiSe@MC; Figure S3: Nitrogen adsorption-desorption isotherms and pore size distribution curve of NiSe@MC; Figure S4: EDS analysis spectrum of Ni-MOF, Ni@MC, and NiSe@MC; Figure S5: XPS survey spectrum of NiSe@MC; Figure S6: SEM image of NiSe@MC electrode after 200 cycling tests at 1 A g^−1^; Table S1: Previously reported NiSe-based analogues and their application in SIBs [35,36,37].
