# Supplementary material for: Metal-Organic Framework-Derived NiSe Embedded into a Porous Multi-Heteroatom Self-Doped Carbon Matrix as a Promising Anode for Sodium-Ion Battery
Source: Nanomaterials (Basel). 2022 Sep 26;12(19):3345. doi: 10.3390/nano12193345 (PMC9565825; doi:10.3390/nano12193345)
Supplement: Supplementary file 1 [file nanomaterials-12-03345-s001.zip › nanomaterials-1904063-supplementary.pdf]

Supporting Information

Metal–Organic Framework-Derived NiSe Embedded into a Porous Multi-Heteroatom Self-Doped Carbon Matrix as a Promising Anode for Sodium-Ion Battery

Xiaoyan Shi †, Lujun Fang †, Handong Peng, Xizhan Deng and Zhipeng Sun *

School of Materials and Energy, Guangdong University of Technology, Guangzhou 510006, China
* Correspondence: zpsunxj@gdut.edu.cn
† These authors contributed equally to this work.

1. Materials and Methods

Preparation of NiSe@MC composites

The Ni-MOFs was synthesized by a typical hydrothermal method. First, the CTP-COOH (399 mg) ligand and $C_4H_6O_4Ni·4H_2O$ (320 mg) were dissolved in 35 mL of DMF, and the mixture was stirred at room temperature for five minutes, and then the light-green precipitate was isolated from the solution by centrifugation and further washed with DMF and THF several times, and it was further dried at 70 °C overnight.

To obtain the Ni@MC, 1 g Ni-MOFs was annealed at 600°C for 2h in Ar atmosphere with a rate of 1 °C·min^{-1}.

To prepare the NiSe@MC, 0.316 g Se power was dissolved in 10 mL 80% $N_2H_4·H_2O$ and stirred for 10 min, then 0.18 g Ni@MC and 50 mL H_2O were added into the solution and stirred for another 30 min. Then, the mixture was transferred into 100 mL Teflon-lined autoclave and maintained at 200 °C for 12 h. The synthesized products were washed with distilled water and ethanol three times. Then, the products were dried at 80 °C for 10 h in a vacuum oven and then annealed at 600 °C for 2 h under Ar atmosphere.

2. Materials characterization

The X-ray diffraction (XRD) patterns, morphologies and the elemental mapping were test on a X-ray diffractometer (Bruker D8 ADVANCE, Cu Kα radiation), field emission scanning electron microscopy (FESEM, JEOL JSM-6700F) and transmission electron microscopy (TEM, FEI Thermo Talos F200S), respectively. Energy dispersive X-ray (EDX) measurements were performed on an FEI Quanta 250 FEG SEM. X-ray photoelectron spectroscopy (XPS) spectra were performed on an X-ray photoelectron spectrometer (Thermo Fisher ESCALAB 250Xi). Thermogravimetric analyzer (Mettler, 3+/1600HT) were used to determine NiSe@MC in the composite. N_2 adsorption/desorption was conducted by using a Micromeritics ASAP2460 instrument. The specific surface area was collected using the Brunauer-Emmett-Teller (BET) method, and the pore size distribution was calculated from the adsorption branch of the isotherm.

3. Electrochemical measurements

The active material, acetylene black and CMC (7:2:1, w/w/w) were mixed with distilled water.) to form a slurry. Then the mixture was uniformly applied onto an Cu foil and vacuum-dried at 60 °C for 12 h. The mass loading of active material was 0.85-1.1 mg cm^{-2}. A glass fiber membrane and Na-metal foil were used as separator and counter electrode. The electrolyte was

$NaPF_6$ soluble in Diglyme，The cell were galvanostatically charged/discharged at battery testing system (Shenzhen Neware Battery Co., China). Both cyclic voltammetry (CV) and electrochemical impedance spectroscopy (EIS) were tested by an electrochemical workstation (CHI760e, China) with a scanning speed of 0.1 mV s^{-1} at 0.01 V~3 V, and in a frequency ranging from 10^{-2} Hz to 10^5 Hz.

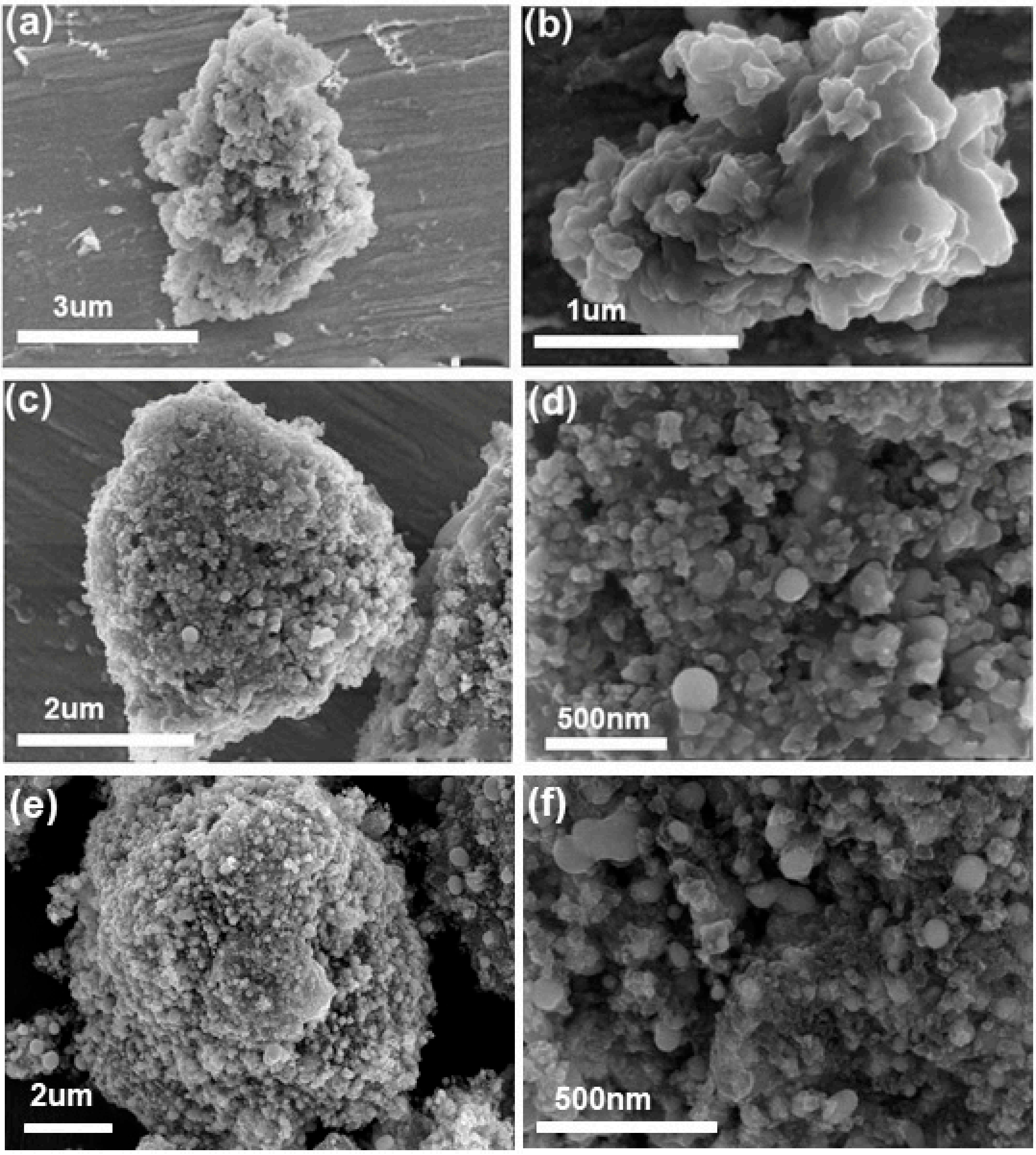

Figure S1. SEM images of Ni-MOFs (**a**,**b**), Ni@MC (**c**,**d**), and NiSe@MC (**e**,**f**).

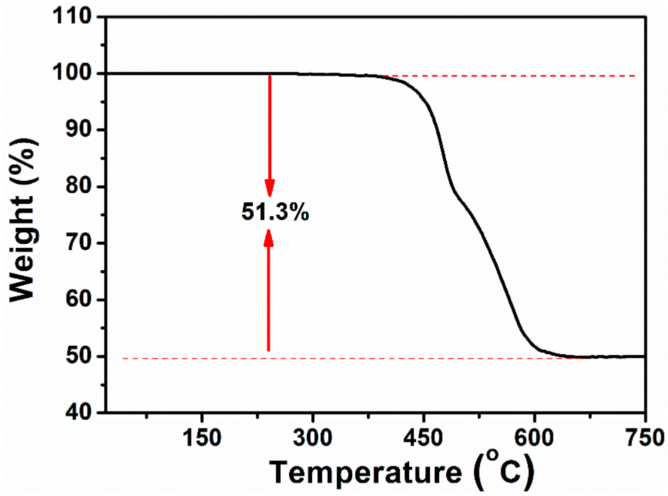

Figure S2. TGA curve of NiSe@MC.

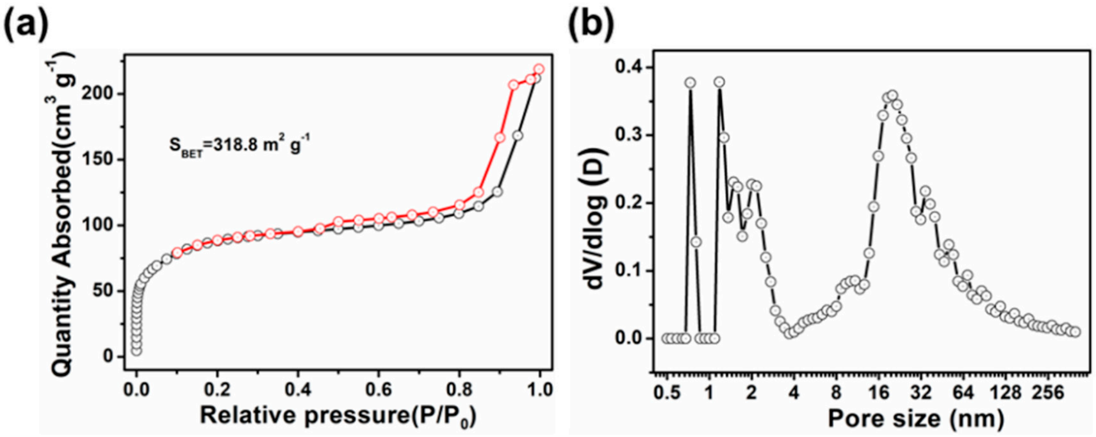

Figure S3. Nitrogen adsorption-desorption isotherms (**a**) and pore size distribution curves (**b**) of NiSe@MC.

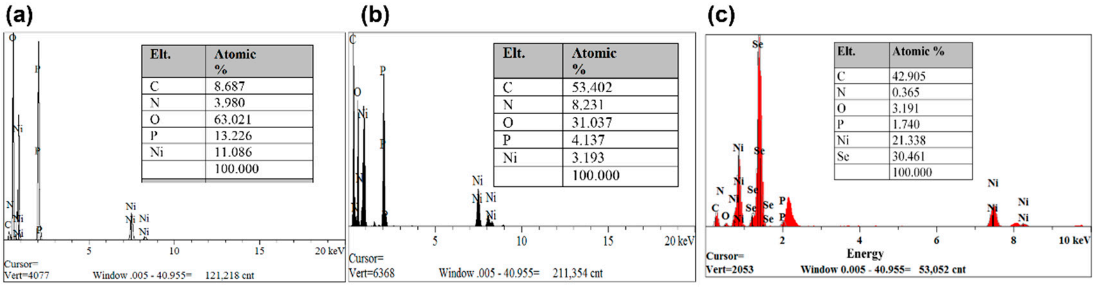

Figure S4. EDS analysis spectrum of Ni-MOF (**a**), Ni@MC (**b**), and NiSe@MC (**c**).

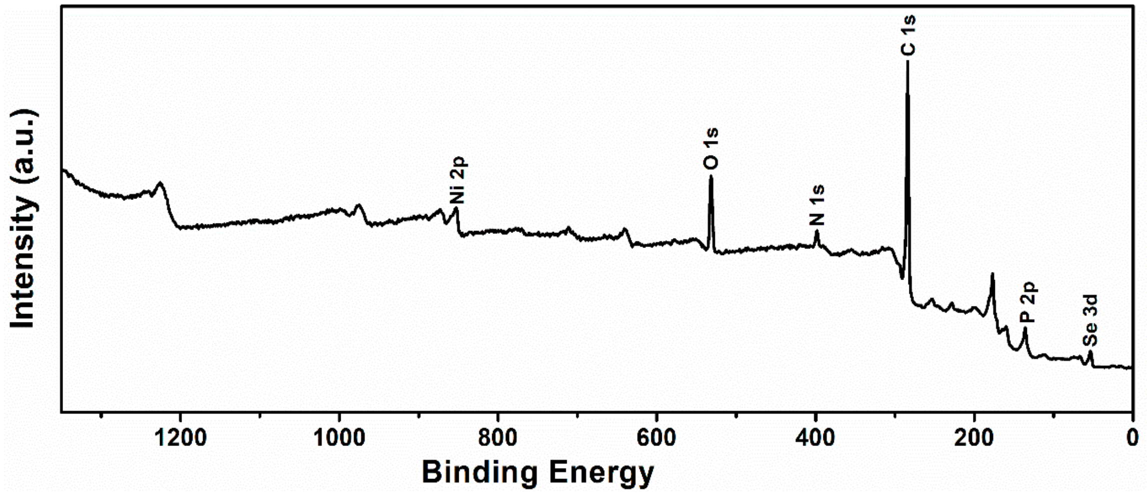

Figure S5. XPS survey spectrum of NiSe@MC.

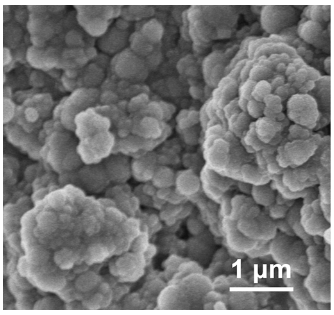

Figure S6. SEM image of NiSe@MC electrode after 200 cycling tests at 1 A g^{-1}.

Table S1. Previously reported NiSe-based analogues and their application in SIBs.

Materials.	Morphology	Voltage window (V vs. Na/Na^+)	Voltage of discharge platforms (V vs. Na/Na^+)	Cycle number	Capacity ($mAh\ g^{-1}$) (A g^{-1})	Ref.
NiSe/rGO	Nanoparticles and nanosheets	0-3	1.0, 1.35	50	378 (0.05)	35
NiSe@C	hollow microspheres	0.5-3.0	1.11, 1.44	100<br>2000	276.4 (0.3)<br>160 (3)	21
NiSe@C@NCNFs	nanofibers	0.01-3.0	0.96	100<br>1000	374 (0.1)<br>164 (2)	36
P–NiSe@C	Particles on the	0.01–3.0	0.99, 1.25	100	598.2	16

	cole pollen				(0.2)	
NiSe/C	core-shell like	0.01–3.0	1.1, 1.2	50	280 (0.1)	37
**NiSe@MC**	**Multi-heteroatom doped carbonaceous NiSe particles**	**0.01-3.0**	**1.46, 1.12, 0.63**	**1000 1200**	**568.9 (1) 227.8 (5)**	**this work**

References

1. Liu, Y.; Wang, X. Reduced Graphene Oxides Decorated NiSe Nanoparticles as High Performance Electrodes for Na/Li Storage. *Materials* **2019**, *12*, 3709.
2. Cu, Q.; Shang, C.; Hu, L.; Zhou, G.; Wang, X. Freestanding carbon nanofibers encapsulating MOF-derived NiSe with in-situ porous carbon protective layer for sodium storage. *Appl. Surf. Sci.* **2022**, *579*, 152181.
3. Zhang, Z.; Shi, X.; Yang, X. Synthesis of core-shell NiSe/C nanospheres as anodes for lithium and sodium storage. *Electrochim. Acta* **2016**, *208*, 238–243.
